# Case report: Central alveolar hypoventilation in a survivor of cardiopulmonary arrest

**DOI:** 10.3389/fneur.2023.1195008

**Published:** 2023-08-03

**Authors:** Fajun Wang, Joseph Darby

**Affiliations:** ^1^Department of Critical Care Medicine, University of Pittsburgh Medical Center, Pittsburgh, PA, United States; ^2^Department of Neurology, Saint Louis University, Saint Louis, MO, United States

**Keywords:** Ondine’s curse, central alveolar hypoventilation, cardiac arrest and brain injuries, nucleus tracti solitarii, dysautonomia

## Abstract

**Introduction:**

Ondine’s curse is a rare respiratory disorder that is characterized by central alveolar hypoventilation (CAH) during sleep. It is most commonly congenital. However, it can also be acquired very rarely. Herein, we report a young survivor who developed CAH following cardiopulmonary arrest.

**Case presentation:**

A 35-year-old man was admitted to the Intensive Care Unit following unwitnessed cardiopulmonary arrest. Following resuscitative interventions, he remained comatose. Early diagnostic testing showed elevated neuronal specific enolase (28.7 ng/ml), absent cortical responses on evoked potential testing and MRI evidence of restricted diffusion in the cerebellum, hippocampi, juxtacortical white matter, superior cerebellar peduncles, dorsal pons, dorsolateral medulla, and upper cervical spinal cord. Ten days following admission, the patient remained comatose and underwent tracheostomy. He subsequently began to emerge from coma but had persistent unexplained hypotension and bradypnea necessitating ongoing vasopressor and respiratory support. Repeat MRI on hospital day 40 revealed residual FLAIR hyperintensities in the medulla within the nucleus tractus solitarius (NTS). After being discharged to long-term acute care facility, he was successfully liberated from mechanical ventilation 70 days post arrest.

**Conclusion:**

We report the first survivor of cardiopulmonary arrest who was complicated by CAH and hypotension with MRI verified ischemic injury to the bilateral NTS regions. Despite this injury, ventilator and vasopressor dependency resolved over a period of 10 weeks. Our case highlighted the essential functions of NTS in regulating the respiratory and cardiovascular systems.

## Introduction

Ondine’s curse is a rare but potentially lethal respiratory disorder that is characterized by central alveolar hypoventilation (CAH) or apnea during sleep. While usually congenital, it can be acquired from neurological disorders. We describe a young man who developed CAH after resuscitation from cardiopulmonary arrest.

## Case description

**Figure 1:**
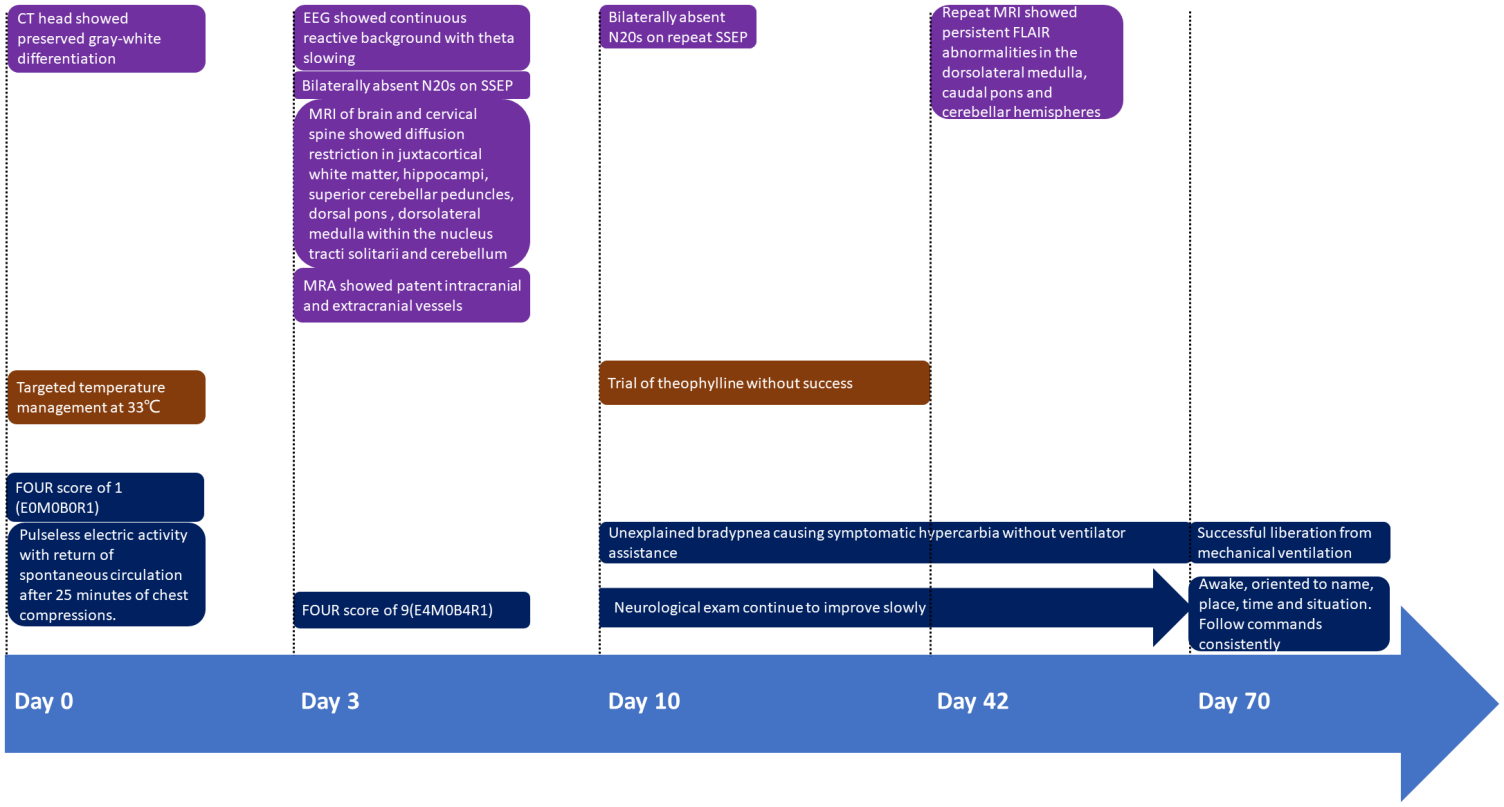
Timeline of presentation and outcome in this case. FOUR Score: Full Outline of UnResponsiveness (FOUR) score of 1 (E0M0B0R1); E, eye response; M, motor response; B, brainstem reflexes; R, respiration pattern.

A nonobese 35-year-old man with history of polysubstance use was transported to the Emergency Department following unwitnessed cardiopulmonary arrest. His initial rhythm was pulseless electric activity with return of spontaneous circulation after 25 min of chest compressions. Initial evaluation revealed an unremarkable electrocardiogram, undetectable blood glucose level and a Full Outline of UnResponsiveness (FOUR) score of 1 (E0M0B0R1). Urine toxicology was positive for cocaine and fentanyl. Neurological examination demonstrated generalized myoclonus and increased muscle tone in the lower extremities with sustained clonus at both ankles. Initial computed tomography (CT) head showed preserved gray-white differentiation and hypodensity in the left cerebellar hemisphere. He was further resuscitated in the intensive care unit (ICU) and subsequently cooled to 33°C for 24 h according to our institutional protocol. Neuron specific enolase was elevated at 28.7 ng/ml. Continuous electroencephalogram (cEEG) showed background suppression ratio of 85% which improved to 10% overnight (Figure 1).

**Figure 2:**
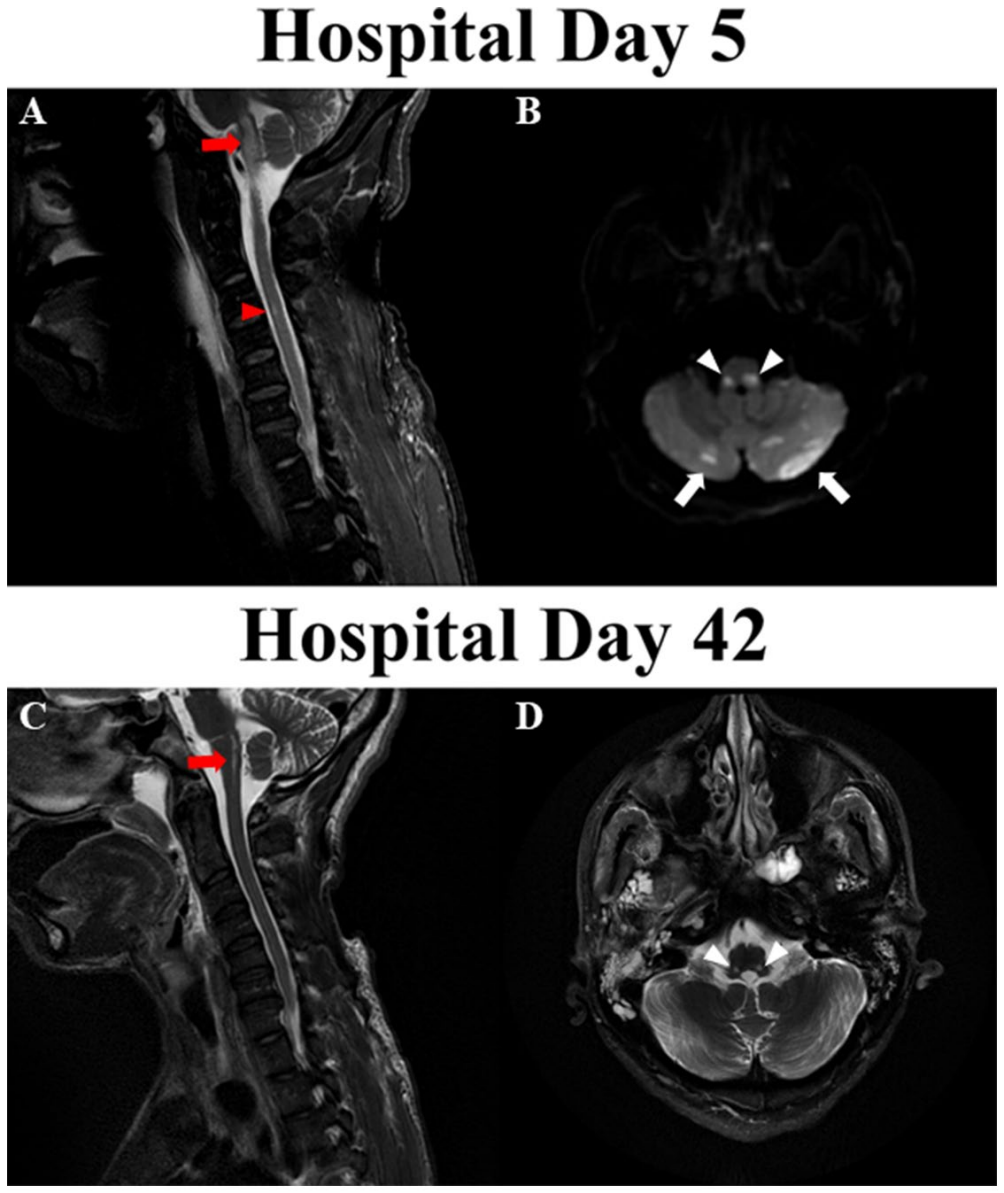
Magnetic resonance imaging of brain and cervical cord. **(A)** Fluid-attenuated inversion recovery (FLAIR) abnormalities in medulla oblongata (red arrows) and ventral aspect of C3-C4 cervical cord (red arrowheads) on hospital day 5. **(B)** Diffusion restriction in dorsolateral medulla (white arrowheads) and bilateral cerebellar hemispheres (white arrows) on hospital day 5. **(C)** Improvement in the FLAIR signal in the medulla oblongata (red arrow) and resolution of FLAIR abnormality in cervical cord on hospital day 42. **(D)** Remnant T2 signal abnormalities in the regions bilateral nucleus tracti solitarii on hospital day 42.

On hospital day 3, his FOUR score improved to 9 (E4M0B4R1) while cEEG revealed continuous reactive background with theta-delta slowing. His eyes opened spontaneously, but he did not react to noxious stimuli. Somatosensory evoked potential (SSEP) revealed bilaterally absent cortical potentials. Given the discrepancy between neurological examination, cEEG and SSEP findings, magnetic resonance imaging (MRI) of brain and cervical spine revealed symmetrical diffusion restriction with surrounding edema in the juxtacortical white matter, hippocampi, superior cerebellar peduncles, dorsal pons, dorsolateral medulla within the nucleus tractus solitarius (NTS), and cerebellum. Punctate hyperintensities were apparent in the left ventral spinal cord at C3-C4 on fluid-attenuated inversion recovery (FLAIR) sequences indicating subacute infarct (Figures 2A,B). There were no vascular abnormalities identified on magnetic resonance angiogram (MRA) of head and neck vessels.

On hospital day 10, repeated SSEP again showed bilaterally absent cortical potentials. After discussion with family, tracheostomy and percutaneous endoscopic gastrostomy tubes were placed. His arousal continued to improve over time and began to track with his eyes and eventually was able to follow commands and communicate. Despite his improving neurological condition, unexplained hypotension requiring vasopressors as well as bradypnea necessitated retention in the ICU. While he was able to breathe voluntarily, his involuntary respiratory rate consistently ranged from between 4 and 6 times per minute with symptomatic hypercarbia developing after variable periods of unassisted breathing. While the administration of pseudoephedrine improved his blood pressure, a trial of theophylline as a respiratory stimulant was ineffective in preventing the development of symptomatic hypercarbia.

On hospital day 42 repeat MRI revealed persistent FLAIR abnormalities in the dorsolateral medulla, caudal pons and cerebellar hemispheres, and resolution of FLAIR abnormalities in the hippocampi, juxtacortical white matters and upper cervical cord (Figures 2C,D). Given his overall improvement in neurological condition and imaging findings, he was transferred to a long term-acute care facility for ongoing ventilator weaning 50 days after hospital admission. Pseudoephedrine was subsequently discontinued with normal blood pressure. He was successfully liberated from mechanical ventilation approximately 70 days after arrest.

## Discussion

Ondine’s Curse was first described by Severinghaus and colleagues in patients who had undergone bilateral spinothalamic tract cordotomies who had retained voluntary respiratory function but poor responsiveness to inhaled carbon dioxide (1). Interchangeably, CAH describes impaired ventilatory function secondary to an underlying neurological disorder affecting sensory, motor or integration pathways of brainstem respiratory centers (1–8).

The neuronal control of respiration is complex and integrated with other autonomic systems. Respiratory control neurons are localized to three main areas within the brainstem: (1) the dorsal respiratory group (mainly inspiratory) located in the dorsal medulla within the NTS; (2) the ventral respiratory group, a column of inspiratory, expiratory and rhythm-generating neurons extending from the first segment of cervical spinal cord to below the facial nuclei; (3) the parabrachial/Kölliker-Fuse complex located in dorsolateral upper pons which controls switching between inspiration and expiration (9). The NTS is also the main area for cardiorespiratory control (9). NTS receives afferent sensory information from peripheral and central chemoreceptors as well as from stretch receptors in the lungs. It then sends information to respiratory motor centers to regulate respiration (10). The NTS also receives afferent information from arterial baroreceptors and cardiac receptors to regulate blood pressure and heart rate with multiple efferent projections to the medulla and pons.

CAH is more frequently congenital than acquired and attributed to a mutation in the PHOX2B transcription factor gene within neurons of the retrotrapezoid nucleus in the ventral respiratory group disrupting central and peripheral chemosensitivity (11). However, traumatic, ischemic, and inflammatory insults to the brainstem can also cause acquired CAH (1–8). Although the respiratory centers receive bilateral reciprocal inputs, patients with unilateral caudal brainstem lesion have been reported to develop CAH (1, 2, 4, 5, 8). Damage to C2 nerve fibers were also implicated in patients developing CAH after high cervical cord injury (12). Moreover, brainstem ischemia or hypoperfusion can also result in acquired CAH (13). In an autopsy report of five patients with acute heart failure and prolonged hypotension, including one patient with cardiopulmonary arrest, bilateral isolated NTS lesions were identified with signs of neuronal excitotoxicity at autopsy (14). The authors proposed that NTS possibly resides in the watershed area of anterior spinal artery (ASA) and posterior inferior cerebellar artery (PICA), which makes them susceptible to ischemic injury during prolonged hypoperfusion. Furthermore, hyperexcitability of NTS neurons in the setting of decreased pH and/or carbon dioxide retention might precipitate secondary injuries.

Herein we report a case of CAH in a young survivor following cardiopulmonary arrest with MRI evidence of bilateral NTS injury. He had CAH with primary respiratory acidosis while preserving the ability of voluntary breathing. His brain MRI showed bilateral symmetrical lesions in the NTS areas, which explained both CAH and dysautonomia with low blood pressure (15). The exact etiology of these lesions might be difficult to pinpoint because his cardiopulmonary arrest was confounded by critical hypoglycemia and substance intoxication. However, here we propose that hypoperfusion from cardiopulmonary arrest is likely the culprit. Firstly, the patient had signs of ischemia in the ASA and PICA territories regardless of patent vessels. Transient drug-induced vasospasm might be another precipitating factor as cocaine is known to cause diffuse vasospasm, even though we did not discover any vascular abnormalities on MRA. Nevertheless, the NTS probably suffered from critical hypoperfusion because it locates in the watershed area of ASA and PICA. Moreover, NTS neurons are hyperexcitable in the setting of acidemia and hypercapnia which might lead to excitotoxicity and exacerbates secondary injuries. Lastly, critical hypoglycemia and drug intoxication might have amplified the excitotoxicity and secondary injuries.

## Conclusion

We report a survivor of cardiopulmonary arrest who was complicated by CAH and hypotension with MRI verified ischemic injury to the bilateral NTS regions. Despite this injury, ventilator and vasopressor dependency resolved over a period of 10 weeks. Our case highlighted the essential functions of NTS in regulating the respiratory and cardiovascular systems.

## Data availability statement

The original contributions presented in the study are included in the article/supplementary material, further inquiries can be directed to the corresponding author.

## Ethics statement

Ethical review and approval was not required for the study on human participants in accordance with the local legislation and institutional requirements. The patients/participants provided their written informed consent to participate in this study. Written informed consent was obtained from the individual(s) for the publication of any potentially identifiable images or data included in this article.

## Author contributions

FW: study concept and design, data acquisition, first draft, and literature review. JD: study concept and design, literature review, and revision of the manuscript for intellectual content. All authors contributed to the article and approved the submitted version.

## Conflict of interest

The authors declare that the research was conducted in the absence of any commercial or financial relationships that could be construed as a potential conflict of interest.

## Publisher’s note

All claims expressed in this article are solely those of the authors and do not necessarily represent those of their affiliated organizations, or those of the publisher, the editors and the reviewers. Any product that may be evaluated in this article, or claim that may be made by its manufacturer, is not guaranteed or endorsed by the publisher.
